# Fractal fractional analysis of non linear electro osmotic flow with cadmium telluride nanoparticles

**DOI:** 10.1038/s41598-022-23182-0

**Published:** 2022-11-23

**Authors:** Saqib Murtaza, Poom Kumam, Attapol Kaewkhao, Naveed Khan, Zubair Ahmad

**Affiliations:** 1 Department of Mathematics, Faculty of Science, King Mongkut’s University of Technology Thonburi (KMUTT), 126 Pracha Uthit Rd., Bang Mod, Thung Khru, Bangkok, 10140 Thailand; 2 Center of Excellence in Theoretical and Computational Science (TaCS-CoE), Faculty of Science, King Mongkut’s University of Technology Thonburi (KMUTT), 126 Pracha Uthit Rd., Bang Mod, Thung Khru, Bangkok, 10140 Thailand; 3 Department of Medical Research, China Medical University Hospital, China Medical University, Taichung, 40402 Taiwan; 4 Data Science Research Center, Department of Mathematics, Faculty of Science, Chiang Mai University, Chiang Mai, 50200 Thailand; 5 Department of Mathematics, City University of Science and Information Technology, Peshawar, Khyber Pakhtunkhwa Pakistan; 6 Dipartimento di Matematica e Fisica, Universit‘a degli Studi della Campania “Luigi Vanvitelli”, 81100 Caserta, Italy

**Keywords:** Mathematics and computing, Nanoscience and technology

## Abstract

Numerical simulations of non-linear Casson nanofluid flow were carried out in a microchannel using the fractal-fractional flow model. The nano-liquid is prepared by dispersing Cadmium Telluride nanoparticles in common engine oil. Using relative constitutive equations, the system of mathematical governing equations has been formulated along with initial and boundary conditions. Dimensionless variables have been used to obtain the non-dimensional form of the governing equations. The fractal-fractional model has been obtained by employing the fractal-fractional operator of the exponential kernel. As the exact solution of the non-linear fractal-fractional model is very tough to find, therefore the formulated model has been solved numerically via the Crank-Nicolson scheme. Various plots are generated for the inserted parameters. From the analysis, it has been observed that a greater magnitude of the electro-kinetic parameter slows down the fluid's velocity. It is also worth noting that the fractional and classical models can also be derived from the fractal-fractional model by taking the parameters tend to zero. From the analysis, it is also observed that in response to 0.04 volume fraction of cadmium telluride nanoparticles, the rate of heat transfer (Nusselt number) and rate of mass transfer (Sherwood number) increased by 15.27% and 2.07% respectively.

## Introduction

Because of its numerous applications in biomechanics and engineering, electro-magneto-hydrodynamics (EMHD) has attracted considerable scientific interest in recent years. EMHD is used in many different applications, including microfluidics, micromechanical systems (MEMS), chemical devices, biomedical devices, biochemical analysis, biomedical diagnostic tools, separation processes, reverse osmosis, and many more. Micropump functions by creating a pressure gradient through a pumping device by using a flow actuation mechanism. These devices are difficult to maintain and rely on moving parts to generate flow. When water or an aqueous solution comes into touch with a charged solid surface, negative charges are generated. Positive ions will be pulled to the surface, whereas negative ions will resist. As a result, an electrical double layer forms, which is a thin layer with a charge imbalance (EDL). When it is parallel to a solid surface, the positively charged EDL moves in the direction of the electromagnetic field. The viscous action causes bulk liquid motion as a result. An electroosmotic flow (EOF) device has been developed for transporting small amounts of liquid in microchannels and capillaries that have proven to be highly profitable. Pressure-driven microchannel transport is superior to EOF due to several advantages. A few examples of electroosmosis are ions-exchange membrane design, biochip manufacturing, microbial fuel cells, non-absorbent polymers in injection systems, corrosion mitigation in civil engineering, nano-bot propulsion for medical use, and many other nano- and micro-scale technological applications. In 1809, Reuss defined the EOF for the first time. Wiedemann (1852) later devised the electro-osmotic flow mathematical theory. The rigorous demands and benefits of EOF devices have led numerous researchers to study EOF with various flow assumptions and fluid models. Industrial, engineering, and biological analyses often require electroosmosis and peristalsis to occur simultaneously. The motion of an electrolyte through a microchannel caused by variable electroosmosis and peristalsis was investigated by Tripathi et al.^1^. By increasing the electrical field parameter, it is possible to increase the maximum average flow rate. To analyze biofluid flow through a microchannel transmitted by a peristaltic wave, Shit et al.^2^ constructed a mathematical model based on the couple-stress fluid model. Their results revealed a significant impact of the electro-osmotic parameter and magnetic field strength on trapping bolus formation. Tripathi et al.^3^ analyzed the EOF of aqueous nanofluids using a peristalsis-generated microchannel. The pressure difference increases when the flow direction changes when an electric field is present in the direction of the flow. The EOF of micropolar fluids was studied by Chaube et al.^4^ by using peristaltic pumping in a microchannel. By applying external electric fields to peristaltic pumps, the paper demonstrated that pumping could be altered^5–10^ also cites related works in this field.

Nature is extremely difficult to mathematically express. Explains the reasons derivatives cannot sufficiently describe some phenomena. Researchers from virtually all fields of science, technology, and engineering have been attracted to non-local operators of differentiation in recent years because of their ability to accommodate more complex natural processes into mathematical equations. These laws have all been proposed as dominant in this field, including the power law, exponential decay law, and generalized Mittag–Leffler law. It was found that Riemann–Liouville and Caputo fractional operators are compatible with power law and non-local and singular kernel types; for non-local and non-singular types, Atangana-Baleanu; and for non-local and non-singular types, Caputo Fabrizio. Unlike power law and exponential decay functions, the kernel Mittag–Leffler function is more general in describing decay. Special types of the Atangana-Baleanu fractional operator include Riemann–Liouville and Caputo-Fabrizio. Fractional calculus is exploited in real-world challenges that respect these numerous mentioned criteria in biomathematics, infectious diseases, fluid mechanics, nanotechnology, mathematical psychology, and chaotic attractors^11–17^. As well as using numerical methods, Arqub and Maayah^18^ computed and solved the integrodifferential equations by using fractional derivatives. Al-Smadi et al.^19^ explore and assess the algebraic-Baleanu sense fuzzy fractional differential equations. Arqub and Maayah^20^ evaluated comprehensively the Bernoulli and Riccati equations utilizing the fractional order derivative concept. Shah et al.^21^ discussed the impact of magnetic field on the double convection problem of fractional viscous fluid over an exponentially moving vertical plate. The authors utilized the Caputo time fractional derivative operator in their analysis. By using the time-fractional derivative operator, Asjad et al.^22^ examined the viscous nanofluid flows over an infinite flat surface. Some other interesting analyses on the uses of fractional derivatives can be seen in^23–25^.

A more effective mathematical differentiation operator is needed for more advanced physical models. Fractional differentiation and fractal derivative are combined into one differential operator by the fractal-fractional operator. Fractal dimension and fractional order are the two orders in the fractional-order derivative's operator. By using the innovative fractal fractional derivative concept, conventional and fractional derivatives are both outperformed. Our examination of the fractal dimension and fractional operator can be done simultaneously when working with fractal-fractional derivatives. A more effective mathematical differentiation operator is needed for more advanced physical models. Fractional differentiation and fractal derivative are combined into one differential operator by the fractal-fractional operator. In fractional-order derivatives, there are two orders in the operator: the fractional-order component and the fractal dimension component. By using the innovative fractal fractional derivative concept, conventional and fractional derivatives are both outperformed. Our examination of the fractal dimension and fractional operator can be done simultaneously when working with fractal-fractional derivatives. Because of its extensive and unique qualities, the fractal fractional operator has piqued the interest of many researchers. Using Caputo-Fabrzio and Atangan-Baleanu fractal-fractional operators, Abro and Atangana^26^ compared convective fluids, using available research. Muhammad Imran Asjad^27^ investigated how to understand the flow between two plates using the fractal-fractional operators of Caputo with a power law kernel. The self-similarities of the 3D chaotic system can be explained by Atangana and Qureshi^28^ using FF derivatives. The dynamics of several unusual attractors were examined using FF derivative operations by Qureshi et al.^29^. With the use of FF derivatives, Gomez-Aguilar and Atangana^30^ studied some other chaotic attractors. The numerical findings presented in Akgul et al.^31^ are for chaotic integrated circuit dynamics with the FF derivative and Mittag–Leffler kernel.

According to a detailed and logical literature review, no such study has been found on the numerical analysis of the non-linear fractal-fractional model of electro-osmotic flow. Therefore, a non-linear unsteady flow of Casson nanofluid in a micro-channel has been considered for investigation. In addition to electro-osmosis and magnetic field, viscous dissipation has also been considered. The fractal-fractional model has been formulated in terms of non-linear partially coupled PDEs and then solved by numerical technique i.e. Crank-Nicolson scheme. From the obtained solutions, various plots are generated in response to inserted parameters and then discussed comprehensively.

## Preliminaries

On Sept. 30, 1695, L'Hopital had written a letter to Leibniz. He asked about a particular notation that was used by Leibniz for nth order derivative that what would be the answer if *n* = 1/2? Leibniz responded that it is a paradox from which, one day, some useful consequences will be drawn^32^. The communication between Leibniz and L'Hopital had given birth to fractional calculus. Fractional calculus is a branch of mathematics that generalizes the idea of conventional integral and derivatives of integer order to non-integer order integral and derivatives. For the last five decades, after the work of Caputo^33^, this field attained great consideration by researchers due to its applications in real-world problems by utilizing the usual initial condition in the application of Laplace transform in contrast to the unusual initial condition in case of Riemann–Liouville fractional derivative operator. The very well-known and much-used fractional operators are Riemann–Liouville and Caputo derivatives. But despite too much significance, these derivatives have some drawbacks to the singular kernel. After the successful application of fractional derivatives in real-world problems, the mathematicians also tried to contribute further to this field. In 2015, Caputo and Fabrizio came to the conclusion that the fractional operators currently in use have several drawbacks that can produce inaccurate results, especially when simulating real-world issues. Some of the experts pointed out that these incorrect results are because of singular kernel. This was a pretty strong argument. This led to the development of the Caputo-Fabrizio fractional derivative (CFFD) operator, which is based on the non-singular exponential kernel provided in ^34^.

1 $$D_{t}^{\alpha } f\left( {y,t} \right) = \frac{N\left( \alpha \right)}{{1 - \alpha }}\int\limits_{0}^{t} {\exp \left( { - \frac{{\alpha \left( {t - \tau } \right)}}{1 - \alpha }} \right)\frac{{\partial f\left( {y,t} \right)}}{\partial \tau }d\tau } ;\,\,\,0 < \alpha < 1$$

here $N\left( \alpha \right)$ is the normalization function and $\frac{N\left( \alpha \right)}{{1 - \alpha }}\exp \left( { - \frac{{\alpha \left( {t - \tau } \right)}}{1 - \alpha }} \right)$ is the non-singular exponential kernel. It is worth mentioning here that integer order calculus has a precise physical explanation and is used in the description of numerous ideas of classical physics and applied mathematics.

It is quite challenging to give a suitable mathematical model with ordinary-order differentiation in the context of fluid dynamics due to the numerous complexities, unknowns, and misleading information. Since they can capture non-localities and some memory effects depending on whether there is a power law, crossover effects, or fading memory, nonlocal operators are typically the best choice for such circumstances. Nevertheless, there are more complex behaviors that could not be replicated with a power law, crossover, and fading memory. Fractal-fractional operators, which were just introduced, will be better mathematical tools to handle such behaviours.

In mechnaics, we always consider the fluid (liquid and air) flow is continous in continous space. the continuum hypothesis holds true for many real-world applications. But it fails in some of the cases, for example, in molecule diffusion phenomena, the water become discontinous and space you assumed not remian as continous space. Therefore, to examine the water diffusion phenomena in discontinous space, the fractal calculus plays key role to analyze the molecules motion. Otherwise, molecule motion becomes completely unpredictable in the frame of the continuum hypothesis.

Recently, a novel idea for differentiation was put forth, where the operator contains two orders: the fractal dimension and the fractional order. Since the concept is relatively new, this kind of differential and integral operator has not yet undergone much study. Atangana demonstrated the relationship between fractal calculus and fractional calculus in^35^ and introduced the fractal-fractional differential and integral operators. Additionally, he provided several novel aspects and numerical estimates of the aforementioned operators with some applicability to actual issues. The mathematical relation between the fractal derivative and fractional derivative is given as^35^:

The Fractal-Fractional derivative of order $\alpha$ in Riemann–Liouville sense with exponential decay kernel is given as:

2 $$^{FF} D_{\tau }^{\alpha ,\beta } f\left( \tau \right) = \frac{M\left( \alpha \right)}{{1 - \alpha }}\frac{d}{{d\tau^{\beta } }}\int\limits_{a}^{\tau } {f\left( \xi \right)\exp \left\{ { - \frac{\alpha }{1 - \alpha }\left( {\tau - \xi } \right)} \right\}d\xi } ,\,\,\,\,\,0 < \alpha ,\,\beta \le 1.$$

## Physical description of the problem

**Figure 1:**
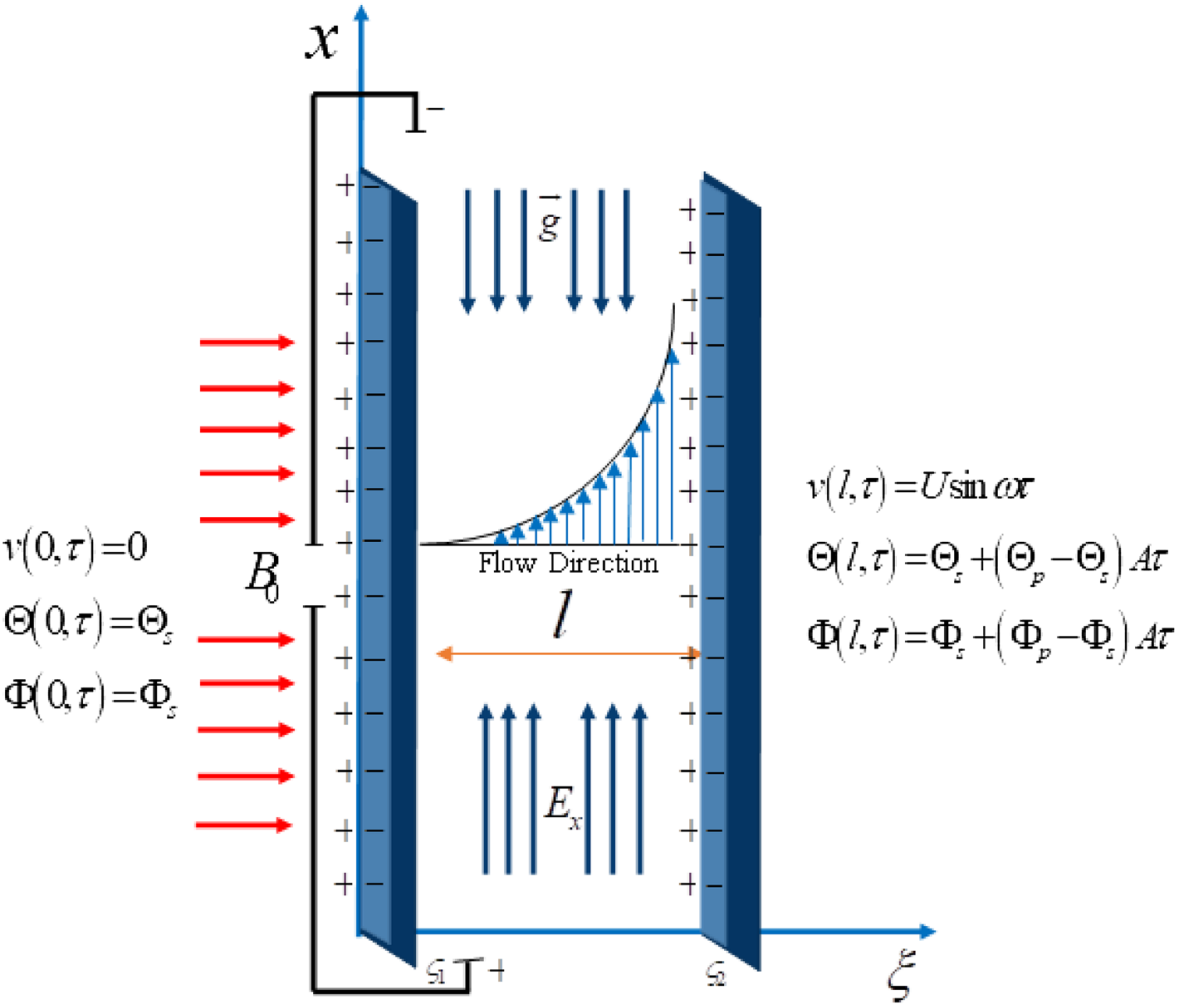
Geometrical illustration of phenomenon.

Fractal fractional electro-osmotic Casson nanofluid flow has been assumed in a microchannel of length $l$. A homogenous mixture of nanofluid has been made by dispersing Cadmium Telluride nanoparticles in engine oil. At the start of analysis ($\tau = 0$), the fluid and the plates are assumed to be static with the ambient temperature $\Theta_{s}$ and ambient concentration $\Phi_{s}$. At the time $\tau > 0$ the left plate is disturbed with the velocity of magnitude $U\sin w\tau$ which transmits the motion in the fluid while the temperature and concentration of the fluid are raised to $\Theta_{s} + \left( {\Theta_{p} - \Theta_{s} } \right)A\tau$ and $\Phi_{s} + \left( {\Phi_{p} - \Phi_{s} } \right)A\tau$ respectively. An illustration of the phenomenon is shown in Fig. 1.

Using the above-stated assumptions, the velocity, thermal, concentration, electric, and magnetic fields are as follows ^36^:

3 $$\left. \begin{gathered} \overrightarrow {V} = \left\{ {v\left( {\zeta ,\,\tau } \right),\,0,\,0} \right\} \hfill \\ \overrightarrow {\Theta } = \,\left\{ {\Theta \left( {\zeta ,\,\tau } \right),\,0,\,0} \right\} \hfill \\ \overrightarrow {\Phi } = \,\left\{ {\Phi \left( {\zeta ,\,\tau } \right),\,0,\,0} \right\} \hfill \\ \overrightarrow {E} = \,\left\{ {E_{x} ,\,0,\,0} \right\} \hfill \\ \overrightarrow {B} = \,\left\{ {0,\,B_{0} ,\,0} \right\} \hfill \\ \end{gathered} \right].$$

Electroosmotic phenomena are present in the velocity field and the constitutive equations for this field are^36^:

4 $$\rho \frac{{d\overrightarrow {V} }}{d\tau } = \overrightarrow {\nabla } .\left( {{{\varvec{\uptau}}}_{ij} } \right) + \rho \overrightarrow {{b_{f} }} .$$

Here $\rho ,\,\,\overrightarrow {V} \,,\,\,{\varvec{\tau}}_{ij}$ and $\rho \overrightarrow {{b_{f} }}$ indicates effective density, velocity, Cauchy stress tensor, and body forces respectively.

5 $$\rho \overrightarrow {{b_{f} }} = \overrightarrow {J} \times \overrightarrow {B} + \rho \overrightarrow {g} + \overrightarrow {E} \rho_{e} .$$

Mathematical form of $\rho_{e}$ is^37^:

6 $$\rho_{e} = - \in k^{2} \varsigma_{1} \left[ {\left( {\frac{{\frac{{\varsigma_{2} }}{{\varsigma_{1} }} - e^{ - kl} }}{{2\sinh \left( {kl} \right)}}} \right)e^{k\zeta } + \left( {1 + \frac{{\frac{{\varsigma_{2} }}{{\varsigma_{1} }} - e^{ - kl} }}{{2\sinh \left( {kl} \right)}}} \right)e^{ - k\zeta } } \right].$$

The rheological equations describing the Casson fluid are given as^38^:

7 $${{\varvec{\uptau}}}_{ij} = \left[ \begin{gathered} \left( {\mu_{\varepsilon } + \frac{{q_{\xi } }}{{\sqrt {2{\varvec{\pi}}_{0} } }}} \right)2e_{ij} ,\,\,{\varvec{\pi}}_{0} > {\varvec{\pi}}_{1} \hfill \\ \left( {\mu_{\varepsilon } + \frac{{q_{\xi } }}{{\sqrt {2{\varvec{\pi}}_{1} } }}} \right)2e_{ij} ,\,\,{\varvec{\pi}}_{0} < {\varvec{\pi}}_{1} \hfill \\ \end{gathered} \right].$$

The value of the cross product of $\overrightarrow {J} \times \overrightarrow {B}$ are calculated from Maxwell equation and generalized Ohm’s law and stated as follow:

8 $$\mathop J\limits^{ \to } \times \mathop B\limits^{ \to } = - \sigma B_{0}^{2} v\left( {\zeta ,\tau } \right).$$

The constitutive form of the thermal and concentration fields are ^39^;

9 $$\rho C_{p} \frac{{\partial \Theta \left( {\zeta ,\tau } \right)}}{\partial \tau } = - \overrightarrow {\nabla } \overrightarrow {{q_{r} }} + \mu \left( {\frac{\partial v}{{\partial \zeta }}} \right)^{2}$$

10 $$\overrightarrow {{q_{\gamma } }} = - K\overrightarrow {\nabla } \Theta \left( {\zeta ,\tau } \right)$$

11 $$\frac{{\partial \Phi \left( {\zeta ,\tau } \right)}}{\partial \tau } = - \overrightarrow {\nabla } \overrightarrow {\eta } ,$$

12 $$\overrightarrow {\eta } = - D\overrightarrow {\nabla } \Phi \left( {\zeta ,\tau } \right).$$

By incorporating Eqs. (3), and (5) to Eq. (8) into Eq. (4), we obtain the constitutive equation for the momentum equation given below:

13 $$\begin{gathered} \rho \frac{{\partial v\left( {\zeta ,\tau } \right)}}{\partial \tau } = \mu \left( {1 + \frac{1}{\chi }} \right)\frac{{\partial^{2} v\left( {\zeta ,\tau } \right)}}{{\partial \zeta^{2} }} - \sigma B_{0}^{2} v\left( {\zeta ,\tau } \right) + \rho g\beta_{\Theta } \left( {\Theta \left( {\zeta ,\tau } \right) - \Theta_{s} } \right) \hfill \\ \,\,\,\,\,\,\,\,\,\,\,\,\,\,\,\,\,\,\,\,\,\,\,\,\,\,\,\,\, + \rho g\beta_{\Phi } \left( {\Phi \left( {\zeta ,\tau } \right) - \Phi_{s} } \right) + E_{x} \rho_{e} \hfill \\ \end{gathered}$$

and in compact form the governing temperature and concentration equations are as follow:

14 $$\rho C_{p} \frac{{\partial \Theta \left( {\zeta ,\tau } \right)}}{\partial \tau } = K\frac{{\partial^{2} \Theta \left( {\zeta ,\tau } \right)}}{{\partial \zeta^{2} }} + \mu \left( {\frac{{\partial v\left( {\zeta ,\tau } \right)}}{\partial \zeta }} \right)^{2}$$

15 $$\frac{{\partial \Phi \left( {\zeta ,\tau } \right)}}{\partial \tau } = D\frac{{\partial^{2} \Phi \left( {\zeta ,\tau } \right)}}{{\partial \zeta^{2} }}$$

associated IBCs^40^:

16 $$\left. \begin{gathered} v\left( {\zeta ,0} \right) = 0,\,\,\,\,\,\,\,\,\,\,\,\,\,\,\,\,\,\,\,\Theta \left( {\zeta ,0} \right) = \Theta_{s} ,\,\,\,\,\,\,\,\,\,\,\,\,\,\,\,\,\,\,\,\,\,\,\,\,\,\,\,\,\,\,\,\,\,\,\,\,\,\Phi \left( {\zeta ,0} \right) = \Phi_{s} , \hfill \\ v\left( {0,\tau } \right) = 0,\,\,\,\,\,\,\,\,\,\,\,\,\,\,\,\,\,\,\,\,\Theta \left( {0,\tau } \right) = \Theta_{s} ,\,\,\,\,\,\,\,\,\,\,\,\,\,\,\,\,\,\,\,\,\,\,\,\,\,\,\,\,\,\,\,\,\,\,\,\,\,\Phi \left( {0,\tau } \right) = \Phi_{s} , \hfill \\ v\left( {l,\tau } \right) = U\sin \omega \tau ,\,\,\,\,\Theta \left( {l,\tau } \right) = \Theta_{s} + \left( {\Theta_{p} + \Theta_{s} } \right){\rm A}\tau ,\,\,\Phi \left( {l,\tau } \right) = \Phi_{s} + \left( {\Phi_{p} + \Phi_{s} } \right){\rm A}\tau \hfill \\ \end{gathered} \right\}.$$

For the nanofluid model, the governing Eqs. (13)–(15) will take the form:

17 $$\begin{gathered} \rho_{nf} \frac{{\partial v\left( {\zeta ,\tau } \right)}}{\partial \tau } = \mu_{nf} \left( {1 + \frac{1}{\chi }} \right)\frac{{\partial^{2} v\left( {\zeta ,\tau } \right)}}{{\partial \zeta^{2} }} - \sigma_{nf} B_{0}^{2} v\left( {\zeta ,\tau } \right) + \left( {\rho \beta_{\Theta } } \right)_{nf} g\left( {\Theta \left( {\zeta ,\tau } \right) - \Theta_{s} } \right) \hfill \\ \,\,\,\,\,\,\,\,\,\,\,\,\,\,\,\,\,\,\,\,\,\,\,\,\,\,\,\,\,\,\,\, + \left( {\rho \beta_{\Phi } } \right)_{nf} g\left( {\Phi \left( {\zeta ,\tau } \right) - \Phi_{s} } \right) + E_{x} \rho_{e} \hfill \\ \end{gathered}$$

18 $$\left( {\rho C_{p} } \right)_{nf} \frac{{\partial \Theta \left( {\zeta ,\tau } \right)}}{\partial \tau } = K_{nf} \frac{{\partial^{2} \Theta \left( {\zeta ,\tau } \right)}}{{\partial \zeta^{2} }} + \mu_{nf} \left( {\frac{{\partial v\left( {\zeta ,\tau } \right)}}{\partial \zeta }} \right)^{2}$$

19 $$\frac{{\partial \Phi \left( {\zeta ,\tau } \right)}}{\partial \tau } = D_{nf} \frac{{\partial^{2} \Phi \left( {\zeta ,\tau } \right)}}{{\partial \zeta^{2} }}.$$

Tables 1 and 2 represent the correlation of base fluid and nanoparticles and their thermo-physical properties respectively.

**Table 1:** Nanofluid correlations are given as^41–46^.

Dynamic viscosity	$\mu_{nf} = \mu_{EO} \left( {1 - \varphi_{CT} } \right)^{ - 2.5}$
The effective density	$\rho_{nf} = \left( {1 - \varphi_{CT} } \right)\rho_{EO} + \varphi_{CT} \rho_{CT}$
Mass diffusion coefficient	$D_{nf} = D_{EO} \left( {1 - \varphi_{CT} } \right)$
Specific heat capacity	$\left( {\rho C_{p} } \right)_{nf} = \left( {1 - \varphi_{CT} } \right)\left( {\rho C_{p} } \right)_{EO} + \varphi_{CT} \left( {\rho C_{p} } \right)_{CT}$
Volumetric thermal expansion	$\left( {\rho \beta_{\rm T} } \right)_{nf} = \left( {\rho \beta_{\rm T} } \right)_{EO} \left( {1 - \varphi_{CT} } \right) + \varphi_{CT} \left( {\rho \beta_{\rm T} } \right)_{CT}$
Volumetric mass expansion	$\left( {\rho \beta_{C} } \right)_{nf} = \left( {\rho \beta_{C} } \right)_{EO} \left( {1 - \varphi_{CT} } \right) + \varphi_{CT} \left( {\rho \beta_{C} } \right)_{CT}$
Electrical conductivity	$\sigma_{nf} = \sigma_{EO} \left[ {1 + \frac{{3\left( {\frac{{\sigma_{CT} }}{{\sigma_{EO} }} - 1} \right)\varphi_{CT} }}{{\left( {\frac{{\sigma_{CT} }}{{\sigma_{EO} }} + 2} \right) - \left( {\frac{{\sigma_{CT} }}{{\sigma_{EO} }} - 1} \right)\varphi_{CT} }}} \right]$
Thermal conductivity	$k_{nf} = k_{EO} \left[ {\frac{{k_{CT} + 2k_{EO} - 2\varphi_{CT} \left( {k_{EO} - k_{CT} } \right)}}{{k_{CT} + 2k_{EO} + \varphi_{CT} \left( {k_{EO} - k_{CT} } \right)}}} \right]$

**Table 2:** Thermo-mechanical characteristics of engine oil and cadmium telluride nanoparticles^39^.

	$\rho \left( {{\text{kg}}/{\text{m}}^{3} } \right)$	$C_{p} \left( {{\text{J}}/{\text{kgK}}} \right)$	$K\left( {{\text{W}}/{\text{mK}}} \right)$	$\beta_{T} \times 10^{ - 5} \left( {{\text{K}}^{ - 1} } \right)$	$\sigma \left( {{\text{Sm}}^{ - 1} } \right)$
Cadmium Telluride	5855	209	7.5	0.00005	0.0000007
Engine oil	863	2048	0.1404	0.00007	0.0000055

Introducing the non-dimensional quantities;

20 $$\begin{gathered} u^{\square } = \frac{v}{{u_{s} }},\,\,\,\,\,\tau^{\square } = \frac{\upsilon }{{l^{2} }}\tau ,\,\,\,\,\,\zeta^{\square } = \frac{\zeta }{l},\,\,\,\,\,{\rm T} = \frac{{\Theta - \Theta_{s} }}{{\Theta_{p} - \Theta_{s} }},\,\,\,\,\,C = \frac{{\Phi - \Phi_{s} }}{{\Phi_{p} - \Phi_{s} }},\,\,\,\,\,k^{\square } = kl, \hfill \\ \Re_{\varsigma } = \frac{{\varsigma_{2} }}{{\varsigma_{1} }},\,\,\,\,\,\lambda^{\square } = \frac{ql}{{k\left( {\Theta_{p} - \Theta_{s} } \right)}},\,\,\,\eta = \frac{jl}{{D\left( {\Phi_{p} - \Phi_{s} } \right)}},\,\,\,A = \frac{v}{{l^{2} }} \hfill. \\ \end{gathered}$$

After inserting the dimensionless quantities along with the nanofluid correlations into the dimensional governing Eqs. (17–19) and ignoring the symbols ($\square$), the governing equations will take the shape of:

21 $$\frac{{\partial u\left( {\zeta ,\tau } \right)}}{\partial \tau } = \chi_{1} \frac{{\partial^{2} u\left( {\zeta ,\tau } \right)}}{{\partial \zeta^{2} }} - M^{*} u\left( {\zeta ,\tau } \right) + Gr^{*} {\rm T}\left( {\xi ,\tau } \right) + Gm^{*} C\left( {\zeta ,\tau } \right) + k^{*2} \left( {\Re_{1} e^{{k^{*} \varsigma }} + \Re_{2} e^{{ - k^{*} \varsigma }} } \right)$$

22 $$\frac{{\partial {\rm T}\left( {\zeta ,\tau } \right)}}{\partial \tau } = \frac{1}{{\chi_{2} }}\frac{{\partial^{2} {\rm T}\left( {\zeta ,\tau } \right)}}{{\partial \zeta^{2} }} + \psi \left( {\frac{{\partial u\left( {\zeta ,\tau } \right)}}{\partial \zeta }} \right)^{2}$$

23 $$\frac{{\partial C\left( {\zeta ,\tau } \right)}}{\partial \tau } = Sc^{*} \frac{{\partial^{2} C\left( {\zeta ,\tau } \right)}}{{\partial \zeta^{2} }}.$$

With non-dimensional conditions:

24 $$\left. \begin{gathered} u\left( {\xi ,0} \right) = 0,\,\,\,\,\,\,\,\,\,\,\,\,\,\,\,\,\,\,\,\,\,\,{\rm T}\left( {\xi ,0} \right) = 0,\,\,\,\,\,\,\,\,\,\,\,\,\,\,C\left( {\xi ,0} \right) = 0, \hfill \\ u\left( {0,\tau } \right) = 0,\,\,\,\,\,\,\,\,\,\,\,\,\,\,\,\,\,\,\,\,\,\,{\rm T}\left( {0,\tau } \right) = 0,\,\,\,\,\,\,\,\,\,\,\,\,\,C\left( {0,\tau } \right) = 0, \hfill \\ u\left( {1,\tau } \right) = \sin \omega \tau ,\,\,\,\,\,\,\,\,\,\,{\rm T}\left( {1,\tau } \right) = \tau ,\,\,\,\,\,\,\,\,\,\,\,\,\,C\left( {1,\tau } \right) = \tau , \hfill \\ \end{gathered} \right\}$$

Following are the dimensionless variables and constants that are produced during calculi:

$$\begin{gathered} u_{s} = \frac{{ \in \xi_{1} E_{x} }}{\mu },\,\,\,\,k,\,\,\,\,k^{\square } ,\,\,\,\,\Re_{\xi } ,\,\,\,\,E_{c} = \frac{{\upsilon_{EO} U}}{{\left( {C_{p} } \right)_{EO} \left( {\Theta_{p} - \Theta_{s} } \right)l}},\,\,M = \frac{{\sigma_{EO} B_{0}^{2} l^{2} }}{{\mu_{EO} }},\,\,Sc = \frac{{\upsilon_{EO} }}{{D_{EO} }} \hfill \\ Gr = \frac{{gl\left( {\beta_{T} } \right)_{EO} \left( {\Theta_{p} - \Theta_{s} } \right)}}{{\upsilon_{EO} \mu_{s} }},\,\,Gm = \frac{{gl\left( {\beta_{C} } \right)_{EO} \left( {\Phi_{p} - \Phi_{s} } \right)}}{{\upsilon \mu_{s} }},\,\,\,\Pr = \frac{{\left( {\mu C_{p} } \right)_{EO} }}{{K_{EO} }},\,\,\,{\text{Re}} = \frac{Ul}{\upsilon }\,\, \hfill \\ \end{gathered}$$

$$\begin{gathered} \chi_{1} = \frac{{a_{2} }}{{a_{1} }}\left( {1 + \frac{1}{\chi }} \right),\,\,\,\chi_{2} = \frac{{\Pr {\text{Re}} b_{1} }}{{b_{2} }},\,\,\Re_{1} = \frac{{\Re_{\xi } - e^{ - k} }}{2\sinh \left( k \right)},\,\,\,\,M^{*} = \frac{{Ma_{3} }}{{a_{1} }},\,\,\psi = \frac{{E_{c} a_{2} }}{{b_{1} }},\,\, \hfill \\ \Re_{2} = 1 - \Re_{1} ,\,\,Gr^{*} = \frac{{Gra_{4} }}{{a_{1} }},\,\,Gm^{*} = \frac{{Gma_{5} }}{{a_{1} }},\,\,a_{1} = \left( {1 - \varphi } \right) + \varphi \frac{{\rho_{EO} }}{{\rho_{CT} }},\,\,a_{2} = \left( {1 - \varphi } \right)^{ - 2.5} , \hfill \\ a_{3} = 1 + \frac{{3\left( {\frac{{\sigma_{CT} }}{{\sigma_{EO} }} - 1} \right)\varphi_{CT} }}{{\left( {\frac{{\sigma_{CT} }}{{\sigma_{EO} }} + 2} \right) - \left( {\frac{{\sigma_{CT} }}{{\sigma_{EO} }} - 1} \right)\varphi_{CT} }},\,\,a_{4} = \left( {1 - \varphi } \right) + \varphi \frac{{\left( {\rho \beta_{\Theta } } \right)_{EO} }}{{\left( {\rho \beta_{\Theta } } \right)_{CT} }},\,\,a_{5} = \left( {1 - \varphi } \right) + \varphi \frac{{\left( {\rho \beta_{\Phi } } \right)_{EO} }}{{\left( {\rho \beta_{\Phi } } \right)_{CT} }}, \hfill \\ b_{1} = \left( {\left( {1 - \varphi } \right) + \varphi \frac{{\left( {\rho C_{p} } \right)_{EO} }}{{\left( {\rho C_{p} } \right)_{CT} }}} \right),\,\,b_{2} = \frac{{k_{CT} + 2k_{EO} - 2\varphi_{CT} \left( {k_{EO} - k_{CT} } \right)}}{{k_{CT} + 2k_{EO} + \varphi_{CT} \left( {k_{EO} - k_{CT} } \right)}},\,\,Sc^{*} = \left( {Sc\left( {1 - \varphi } \right)} \right)^{ - 1} . \hfill \\ \end{gathered}$$

## Discretization of the fractal-fractional model

To simplify the expressions (21)–(23) by employing the fractal-fractional operator of the exponential kernel^47^, Eqs. (21)–(23) will take the form:

25 $$^{FF} \wp_{\tau }^{\alpha ,\beta } u\left( {\zeta ,\tau } \right) = \chi_{1} \frac{{\partial^{2} u\left( {\zeta ,\tau } \right)}}{{\partial \zeta^{2} }} - M^{*} u\left( {\zeta ,\tau } \right) + Gr^{*} {\rm T}\left( {\zeta ,\tau } \right) + Gm^{*} C\left( {\zeta ,\tau } \right) + k^{*2} \left( {\Re_{1} e^{{k^{*} \zeta }} + \Re_{2} e^{{ - k^{*} \zeta }} } \right)$$

26 $$^{FF} \wp_{\tau }^{\alpha ,\beta } {\rm T}\left( {\zeta ,\tau } \right) = \chi_{2} \frac{{\partial^{2} {\rm T}\left( {\zeta ,\tau } \right)}}{{\partial \zeta^{2} }} + \psi \left( {\frac{\partial u}{{\partial \zeta }}} \right)^{2}$$

27 $$^{FF} \wp_{\tau }^{\alpha ,\beta } C\left( {\zeta ,\tau } \right) = Sc^{*} \frac{{\partial^{2} C\left( {\zeta ,\tau } \right)}}{{\partial \zeta^{2} }}.$$

Here $^{FF} \wp_{\tau }^{\alpha ,\beta }$ is the fractal-fractional operator of the exponential kernel^47^,

28 $$^{FF} \wp_{\tau }^{\alpha ,\beta } \varpi \left( \tau \right) = \frac{N\left( \alpha \right)}{{1 - \alpha }}\frac{d}{{d\tau^{\beta } }}\int\limits_{0}^{\tau } {\varpi \left( \zeta \right)\exp \left\{ { - \frac{\alpha }{1 - \alpha }\left( {\tau - \zeta } \right)} \right\}d\zeta } ,\,\,\,\,\,0 < \alpha ,\,\beta \le 1,$$

where $$N\left( 0 \right) = N\left( 1 \right) = 1.$$

For some positive integer N, the grid size in time for the finite difference technique is defined by^47^:

$$\lambda = \frac{1}{N}.$$

The grid points in the time interval $\left[ {0,T} \right]$ are labeled $\tau_{n} = n\lambda$ with $0 \le n \le N.$ The value of the function $\varpi$ at the grid point $\varpi_{i}^{j} = \varpi \left( {\zeta_{i} ,\tau_{j} } \right)$.

A discrete approximation to the Fractal-Fractional operator can be obtained by simple quadrature formula as follows:

29 $$^{FF} \wp_{\tau }^{\alpha ,\beta } \varpi \left( {\tau_{j} } \right) = \frac{N\left( \alpha \right)}{{1 - \alpha }}\frac{d}{{d\tau_{j}^{\beta } }}\int\limits_{0}^{{\tau_{j} }} {\varpi \left( \zeta \right)\exp \left\{ { - \frac{\alpha }{1 - \alpha }\left( {\tau_{j} - \zeta } \right)} \right\}d\zeta } ,\,\,\,\,\,0 < \alpha ,\,\beta \le 1.$$

Which implies:

30 $$^{FF} \wp_{\tau }^{\alpha ,\beta } \varpi \left( {\tau_{j} } \right) = \frac{N\left( \alpha \right)}{{1 - \alpha }}\beta \tau^{\beta - 1} \sum\limits_{j = 1}^{m} {\int\limits_{{\left( {j - 1} \right)k}}^{jk} {\left( {\frac{{\varpi_{i}^{k + 1} - \varpi_{i}^{k} }}{\lambda } + O\left( \lambda \right)} \right)\exp \left\{ { - \frac{\alpha }{1 - \alpha }\left( {\tau_{j} - \zeta } \right)} \right\}d\zeta } } ,$$

equivalently to:

31 $$^{FF} \wp_{\tau }^{\alpha ,\beta } \varpi \left( {\tau_{j} } \right) = \frac{N\left( \alpha \right)}{{1 - \alpha }}\beta \tau^{\beta - 1} \sum\limits_{j = 1}^{m} {\left( {\frac{{\varpi_{i}^{j + 1} - \varpi_{i}^{j} }}{\lambda } + O\left( \lambda \right)} \right)\int\limits_{{\left( {j - 1} \right)k}}^{jk} {\exp \left\{ { - \frac{\alpha }{1 - \alpha }\left( {\tau_{j} - \zeta } \right)} \right\}d\zeta } } .$$

This leads us to the final discretized form^47^:

32 $$^{FF} \wp_{\tau }^{\alpha } \varpi \left( {\zeta ,\tau } \right) = \beta \tau^{\beta - 1} \frac{N\left( \alpha \right)}{\alpha }\left\{ {\frac{{\varpi_{i}^{j + 1} - \varpi_{i}^{j} }}{\lambda } + \sum\limits_{j = 1}^{m} {\left( {\frac{{\varpi_{i}^{j + 1 - m} - \varpi_{i}^{j - m} }}{\lambda } + O\left( \lambda \right)} \right)} } \right\}\delta_{j,m} ,$$

here $$\delta_{j,m} = erf\left\{ {\frac{\alpha j}{{1 - \alpha }}\left( {m - j} \right)} \right\} - erf\left\{ {\frac{\alpha j}{{1 - \alpha }}\left( {m - j + 1} \right)} \right\}$$,

Similarly, by employing the procedure given in^48^, first and second-order classical derivatives can be discretized in the following form:

33 $$\frac{{\partial \varpi \left( {\zeta ,\tau } \right)}}{\partial \zeta } = \frac{1}{2}\left( {\frac{{\varpi_{i + 1}^{j + 1} - \varpi_{i - 1}^{j + 1} }}{2h} + \frac{{\varpi_{i + 1}^{j} - \varpi_{i - 1}^{j} }}{2h}} \right)$$

34 $$\frac{{\partial^{2} \varpi \left( {\zeta ,\tau } \right)}}{{\partial \zeta^{2} }} = \left\{ {\frac{{\left( {\varpi_{i + 1}^{j + 1} - 2\varpi_{i}^{j + 1} + \varpi_{i - 1}^{j + 1} } \right) + \left( {\varpi_{i + 1}^{j} - 2\varpi_{i}^{j} + \varpi_{i - 1}^{j} } \right)}}{{2h^{2} }}} \right\} + O\left( \tau \right).$$

Using discretization (32)–(34), Eqs. (25)–(27) become;

35 $$\frac{N\left( \alpha \right)}{\alpha }\left\{ \begin{gathered} \frac{{\varpi_{i}^{j + 1} - \varpi_{i}^{j} }}{\lambda } \hfill \\ + \sum\limits_{j = 1}^{m} {\left( {\frac{{\varpi_{i}^{j + 1 - m} - \varpi_{i}^{j - m} }}{\lambda }} \right)} \hfill \\ \end{gathered} \right\}\delta_{j,m} = \beta t^{\beta - 1} \left\{ \begin{gathered} \chi_{1} \left\{ {\frac{{\left( {u_{i + 1}^{j + 1} - 2u_{i}^{j + 1} + u_{i - 1}^{j + 1} } \right)}}{{2h^{2} }} + \frac{{\left( {u_{i + 1}^{j} - 2u_{i}^{j} + u_{i - 1}^{j} } \right)}}{{2h^{2} }}} \right\} \hfill \\ - \frac{1}{2}\left( {M^{*} \left( {u_{i}^{j + 1} + u_{i}^{j} } \right) + Gr^{*} \left( {{\rm T}_{i}^{j + 1} + {\rm T}_{i}^{j} } \right) + Gm^{*} \left( {C_{i}^{j + 1} + C_{i}^{j} } \right)} \right) \hfill \\ + k^{*2} \left( {\left( {\Re_{1} + \Re_{2} } \right)\left( {e^{ - kj} \left( {1 - e^{ - k} } \right)} \right)} \right) \hfill \\ \end{gathered} \right\},$$

36 $$\frac{N\left( \alpha \right)}{\alpha }\left\{ {\frac{{{\rm T}_{i}^{j + 1} - {\rm T}_{i}^{j} }}{\lambda } + \sum\limits_{j = 1}^{m} {\left( {\frac{{{\rm T}_{i}^{j + 1 - m} - {\rm T}_{i}^{j - m} }}{\lambda }} \right)} } \right\}\delta_{j,m} = \beta \tau^{\beta - 1} \left\{ \begin{gathered} \chi_{2} \left\{ {\frac{{\left( {{\rm T}_{i + 1}^{j + 1} - 2{\rm T}_{i}^{j + 1} + {\rm T}_{i - 1}^{j + 1} } \right)}}{{2h^{2} }}} \right\} \hfill \\ + \chi_{2} \left\{ {\frac{{\left( {{\rm T}_{i + 1}^{j} - 2{\rm T}_{i}^{j} + {\rm T}_{i - 1}^{j} } \right)}}{{2h^{2} }}} \right\} \hfill \\ + \psi \frac{1}{2}\left( {\frac{{u_{i + 1}^{j + 1} - u_{i - 1}^{j + 1} }}{2h} + \frac{{u_{i + 1}^{j} - u_{i - 1}^{j} }}{2h}} \right)^{2} \hfill \\ \end{gathered} \right\}$$

37 $$\frac{N\left( \alpha \right)}{\alpha }\left\{ {\frac{{C_{i}^{j + 1} - C_{i}^{j} }}{\lambda } + \sum\limits_{j = 1}^{m} {\left( {\frac{{C_{i}^{j + 1 - m} - C_{i}^{j - m} }}{\lambda }} \right)} } \right\}\delta_{j,m} = \beta \tau^{\beta - 1} \left\{ \begin{gathered} Sc^{*} \left\{ {\frac{{\left( {C_{i + 1}^{j + 1} - 2C_{i}^{j + 1} + C_{i - 1}^{j + 1} } \right)}}{{2h^{2} }}} \right\} \hfill \\ + Sc^{*} \left\{ {\frac{{\left( {C_{i + 1}^{j} - 2C_{i}^{j} + C_{i - 1}^{j} } \right)}}{{2h^{2} }}} \right\} \hfill \\ \end{gathered} \right\}.$$

Consider that $\zeta_{i} = ih,$ $0 \le i \le M$ with $Mh = 1$ and $\tau_{n} = n\lambda$, $0 \le n \le N.$

## Nusselt and Sherwood number

Nusselt and Sherwood numbers are given as:

38 $$Nu = - \frac{{k_{nf} }}{{k_{EO} }}\left. {\frac{{\partial {\rm T}}}{\partial \zeta }} \right|_{\zeta = 0}$$

39 $$S_{h} = - D_{nf} \left( {\frac{\partial C}{{\partial \zeta }}} \right)_{\zeta = 0}$$

## Graphical analysis

In this section, the impact of inserted parameters on fluid flow will discuss comprehensively. In the presence of electro-osmosis, magnetic field, and viscous dissipation effects, a nonlinear fractal fractional model of Casson nanofluid has been investigated. The solution of the mathematical model has been obtained by the numerical scheme.

**Figure 2:**
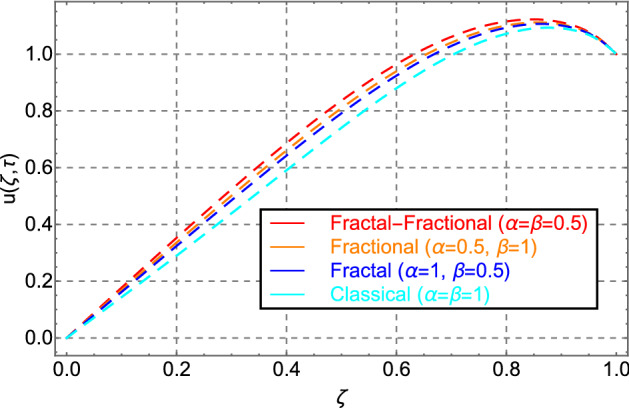
Comparison of velocity distribution for fractal-fractional, fractal, fractional and classical order.

Figure 2 shows the comparison between fractal-fractional, fractional, and classical model behavior. Figure shows that the fractal-fractional model has key significant memory impact than the fractional and classical models. This feature of the fractal-fractional model is due to the extra fractal dimension. Because of the higher memory impact, the FF model is more realistic and applicable to real-world situations. Moreover, the fractal-fractional parameter allows experimentalists to compare their experimental work with one of the best-suited fluid layers. A fractional and classical model can also be derived from the fractal fractional model by taking the parameter $\beta \to 0$ and $\alpha ,\,\beta \to 0$.

**Figure 3:**
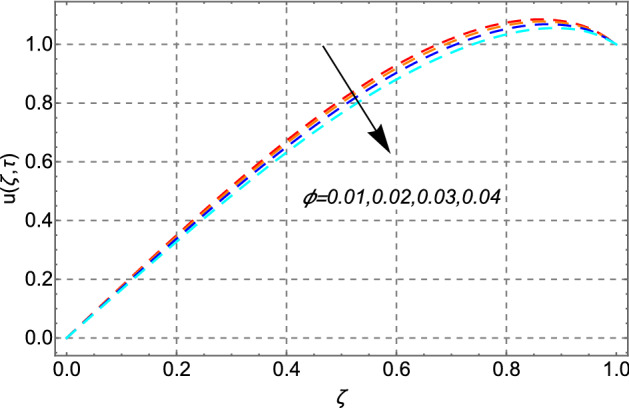
Impact of volume fraction of Cadmium Telluride $\phi$ on velocity distribution.

Change in the velocity field in response to the dispersion of Cadmium telluride nanoparticles in the fluid has been plotted in Fig. 3. From the figure it can be easily analyzed that as the concentration level of cadmium telluride increases the strength of the viscous layer which consequently decrease the fluid motion in a micro channel. From this result, it can be deduced that the lubrication properties of regular engine oil can be improved efficiently by using cadmium telluride particles.

**Figure 4:**
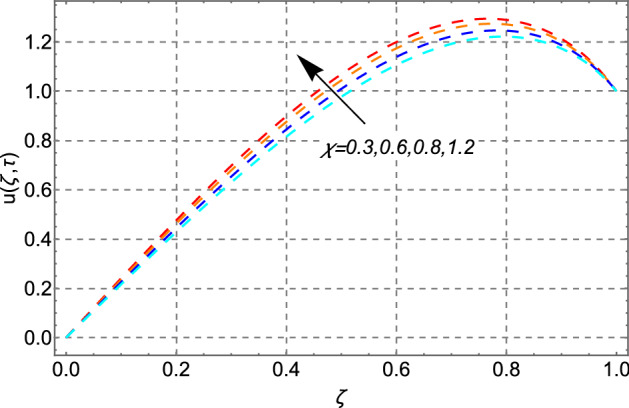
Impact of Casson fluid parameter $\chi$ on velocity distribution.

Figure 4 has been plotted for the influence of the Casson fluid parameter $\chi$ on the fluid’s velocity. From the figure, it is very clear that the increasing magnitude of $\chi$ increases the fluid’s motion in a microchannel. This decreasing trend in the velocity field is due to a decrease in viscosity as the Casson parameter increases and approaches to infinity the Casson fluid is reduced to viscous fluid.

**Figure 5:**
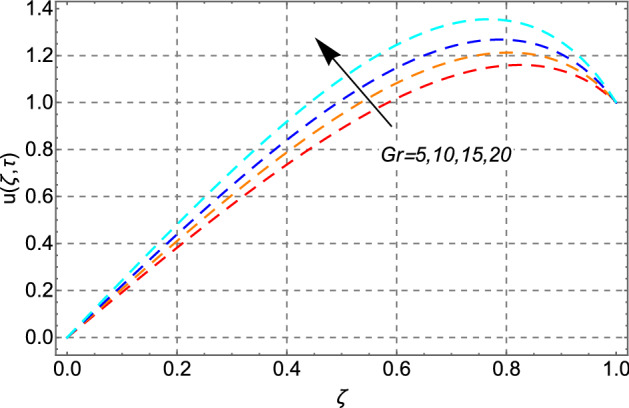
Impact of thermal Grashof number *Gr* on velocity distribution.

The upshots of $Gr$ on the velocity field of engine oil have been plotted in Fig. 5. Increasing behavior has been noticed for the increasing values of *Gr*. Physically, this trend is true because the greater magnitude of *Gr* weakens the boundary layer of the fluid and produces bouncy forces in the fluid. Due to these effects, the fluid motion accelerates.

**Figure 6:**
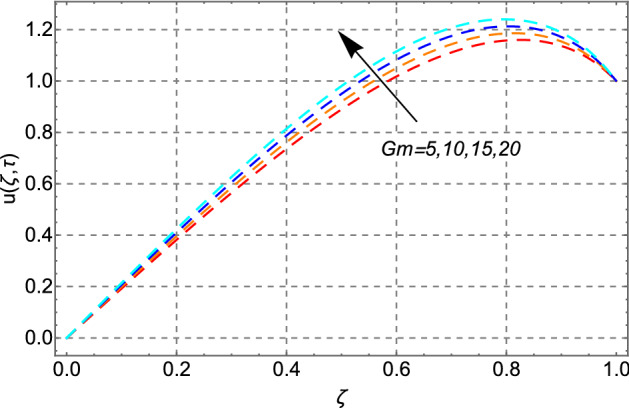
Impact of mass Grashof number *Gm* on velocity distribution.

The upshots of $Gm$ on the velocity field of engine oil have been plotted in Fig. 6. The same trend is noticed as seen in Fig. 5. This trend in the velocity profile is true because as the magnitude of *Gm* increases, the concentration level in the fluid also increases which consequently accelerate fluid’s motion.

**Figure 7:**
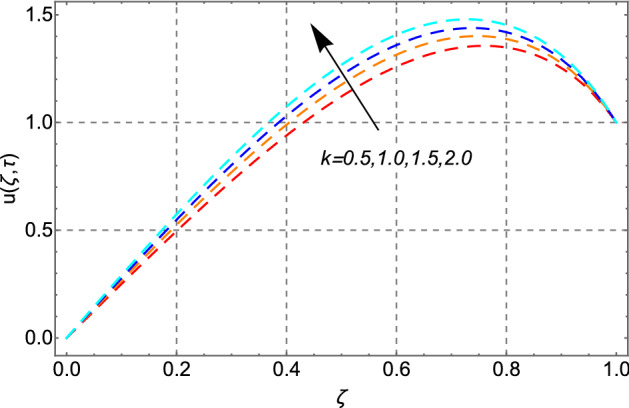
Impact of electro kinetic parameter *k* on velocity distribution.

The impact of electro-kinetic parameter *k* on the fluid’s velocity field has been drawn in Fig. 7. Increasing profile of the velocity has been reported in the response to higher values of electro-kinetic parameter *k.* Greater value of *k* makes the electric double layer thin as a result the drag forces in the fluid become less and consequently fluid’s motion accelerates.

**Figure 8:**
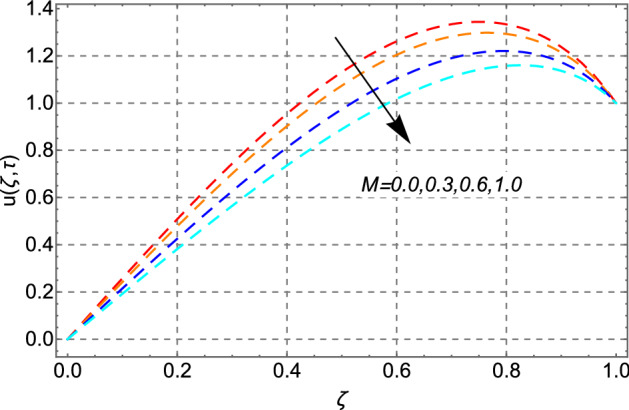
Impact of magnetic parameter *M* on velocity distribution.

The impact of magnetic parameter *M* on the velocity profile has been drawn in Fig. 8. Decreasing profile of the velocity of engine oil has been noticed for the larger values of *M*. This result is quite clear because increasing value of *M* generates drag forces (Lorentz force) in the fluid which retards the fluid motion and as a result, decreasing trend in the profile of velocity has been observed.

**Figure 9:**
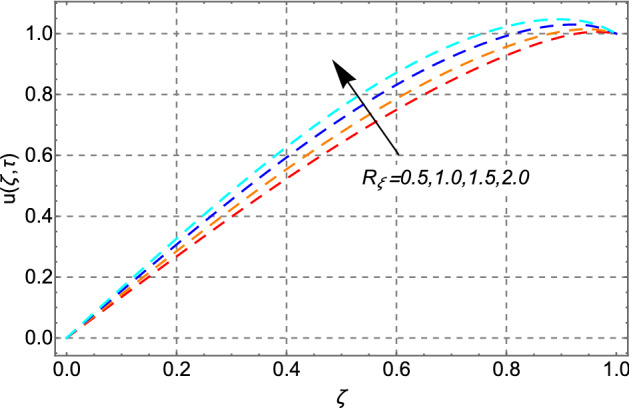
Impact of zeta potential ratio $R_{\xi }$ on velocity distribution.

Figure 9 reveals the upshots of asymmetric zeta potential $R_{\xi }$ on the velocity field. Zeta potential of the plates is related to the magnetic charges in the fluid. So, by increasing the magnitude of $R_{\xi }$, the magnitude of the drag forces in the fluid enhances which consequently thins the EDL and as a result fluid moves fast.

**Figure 10:**
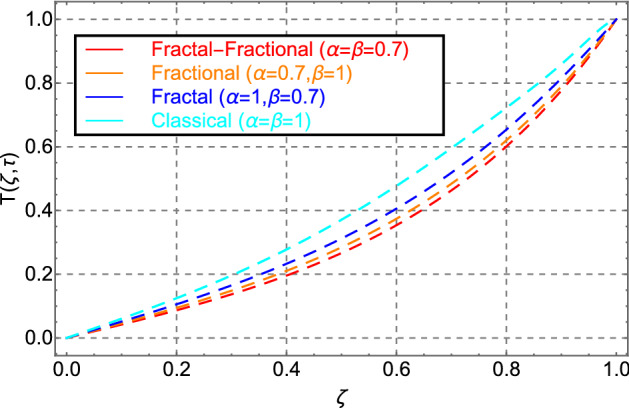
Comparison of temperature distribution for fractal-fractional, fractal, fractional and classical order.

**Figure 11:**
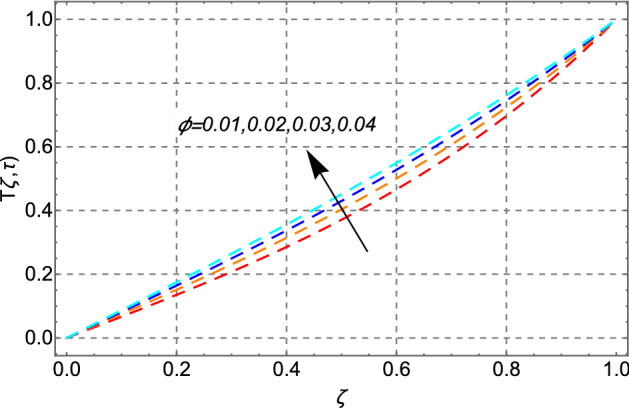
Impact of volume fraction of cadmium telluride nanoparticle $\phi$ on temperature distribution.

Figure 10 shows the same trend for fractal, fractional, fractal-fractional, and classical behavior as shown in Fig. 2. Figure 11 shows the change in thermal energy distribution in response to the volume fraction of cadmium telluride in engine oil. The sketch indicates that when the amount of cadmium telluride nanoparticles increases, the thermal field exhibits rising variation in the profile. This effect is caused by the increased strength of viscous forces in the fluid because of the addition of cadmium telluride nanoparticles to the transformer oil.

**Figure 12:**
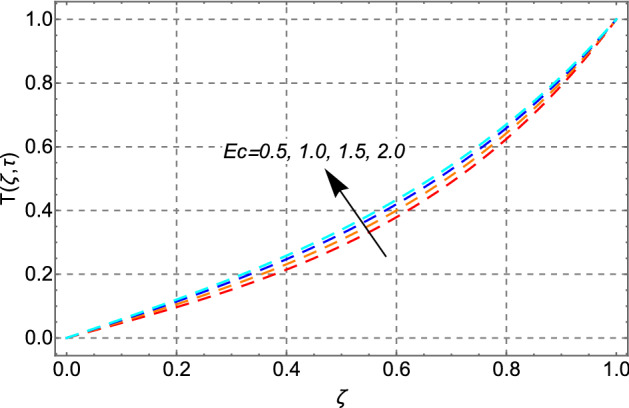
Impact of Eckert number *Ec* on temperature distribution.

**Figure 13:**
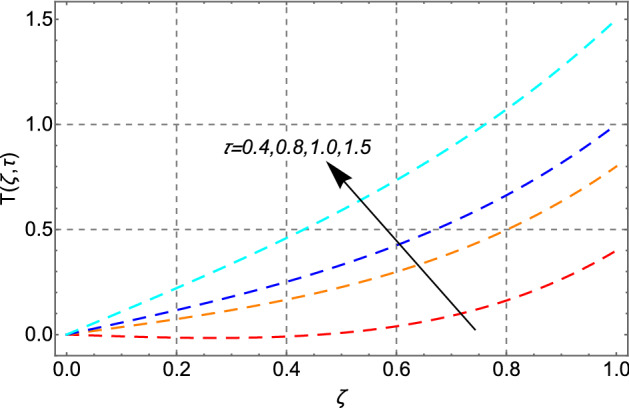
Impact of time parameter $\tau$ effect on temperature distribution.

Figure 12 depicts the effect of the Eckert number $Ec$ on heat transfer. The temperature profile shows a rinsing trend as the magnitude of $Ec$ increases. This is physically valid since $Ec$ denotes the ratio of the boundary layer's kinetic energy to its enthalpy difference. Because a positive $Ec$ indicates heat transfer from the plate to the fluid, increasing dissipation of viscous heat results in a rise in the fluid’s temperature. Figure 13 depicts the effect of time on the thermal field. The graphic shows that the thermal field steadily increases with time.

**Figure 14:**
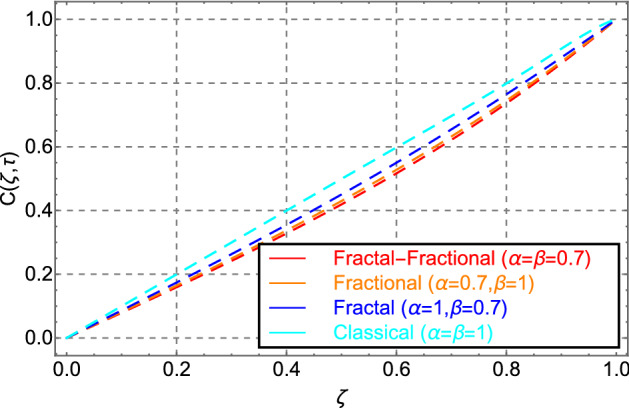
Comparison of Concentration distribution for fractal-fractional, fractal, fractional and classical order.

Figure 14 shows how fractal, fractal-fractional, fractional, and classical order affects concentration field variations. In terms of the memory effect, Figs. 2 and 10 show the same pattern.

**Figure 15:**
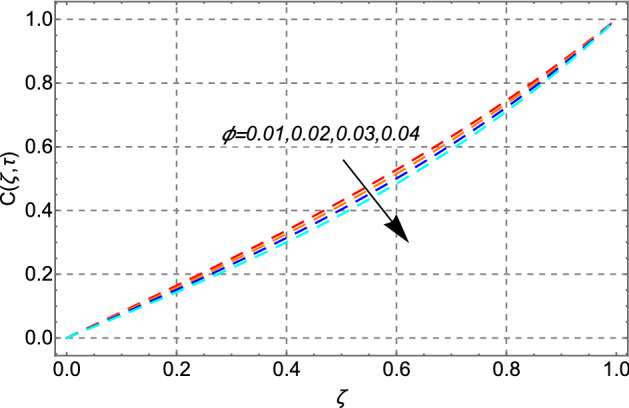
Impact of volume fraction of cadmium telluride nanoparticles $\phi$ on concentration distribution.

The behavior of the concentration profile in response to the volume fraction of Cadmium telluride nanoparticles in conventional engine oil is depicted in Fig. 15. Figure 15 depicts an increasing shift in concentration profile in response to greater values of. This is because greater values $\phi$ of increase the degree of viscous forces in the fluid, causing the concentration profile of the fluid to rise.

Tables 3 and 4 presents the variation in heat and mass transfer against volume fraction. Table 3 reveals that increasing volume fraction, heat transfer increases while mass transfer decreases for larger values of volume fraction $\phi$ which is shown in Table 4. From table, it can be seen that heat transfer enhances up to 15.27% by adding 4% ($\phi = 0.04$) of cadmium telluride nanoparticles in the regular engine oil. Similarly, mass transfer decreases up to 2.07% when adding 4% of cadmium telluride nanoparticles in the base fluid Engine oil.

**Table 3:** Nusselt number for EO based-Cadmium Telluride.

$\phi$	*Nu*	Heat transfer enhancement
0.00	2.763	–
0.01	2.895	3.47%
0.02	2.982	7.93%
0.03	3.086	11.69%
0.04	3.185	15.27%

**Table 4:** Sherwood number for EO-based Cadmium Telluride.

$\phi$	$S_{h}$	Decrease in mass distribution
0.00	3.287	–
0.01	3.268	0.58%
0.02	3.252	1.06%
0.03	3.237	1.52%
0.04	3.219	2.07%

## Concluding remarks

The non-linear fractal-fractional model of electro osmotic Casson nanofluid model was investigated via numerical approach. The investigation was carried out in the presence of a viscous dissipation effect. The mathematical model was first made non-dimensional using non-dimensional quantities, and then it was changed into a fractal-fractional model via the Caputo-Fabrizio derivative operator. The solution of transomed fractal-fractional model was obtained by numerical scheme (Crank–Nicolson). Graphs have been plotted and illustrate comprehensively with physical arguments. The following are the major findings from the analysis:

- Fractal-fractional model is the generalization of both classical and fractional model with crossover behavior, memory effect and fractal characteristics all at once.
- The fractal-fractional model is reduceable to fractional order model by keeping $\beta \to 1$ and also reduceable to classical/integer order model by taking $\alpha \to 1$ and $\beta \to 1$.
- As the parameter $Gr,\,\,Gm,\,\,k$ and $\Re_{\xi }$ increase, the velocity field increases, while velocity profile decreases for $\phi \,\,Sc,\,\,M$ and $\chi$.
- Increasing behavior are observed in the temperature and concentration field in response to $\phi$.
- The rate of heat transfer significantly increased up to 15.27% when the volume fraction of cadmium telluride nanoparticles reached to 0.04.
- The rate of mass transfer decreased up to 2.07% when the volume fraction of cadmium telluride nanoparticles reached to 0.04.

This work can be extended in different directions by considering different Newtonian and non-Newtonian fluid models. This model can also be considered with different physical initial and boundary conditions and different geometries such as spherical, cylindrical coordinates or flow over a thin needle, circular disk and stretching/shrinking sheet. There is also a need to generalize the existing models by considering fractional or fractal-fractional differential operators of different kernels such as power decay, exponential or Mittage-Leffler kernel to find some hidden characteristics of the fluid flow. This model can be considered for different base fluids, nanofluids and hybrid nanofluids in order to study various applications in science, engineering and renewable energy.

## Data Availability

The authors confirm that the data supporting the findings of this study are available with the article. Raw data that support the findings of this study are available from the corresponding author, upon reasonable request.

## List of symbols

${\varvec{\pi}} = e_{ij} ,e_{ij} :\left( {i,j} \right) -$: Deformation rate components
${\varvec{\pi}}$: Product based on the non-Newtonian fluid
$q_{\xi }$: Yield stress of the fluid
$$\Theta$$: Fluid’s temperature
$$q_{\gamma }$$: Heat flux
$$\Phi$$: Fluid’s concentration
$$\mu$$: Dynamic viscosity
$\beta_{\Theta }$: Thermal expansion coefficient
$\chi$: Casson fluid parameter
$u_{s}$: Helmholtz-Smoluchowski velocity
$\Re_{\xi }$: Ratio of the zeta potential
$$M$$: Magnetic number
$Gm$: Mass grashof number
$$\Pr$$: Prandtl number
_IBCs_: Initial and boundary conditions
$$h$$: Space length
$$\beta$$: Fractal dimension
$\overrightarrow {J} \times \overrightarrow {B}$: Vector product of electric current and magnetic field
${\varvec{\pi}}_{1}$: Critical value for the product
${\varvec{\mu}}_{\varepsilon }$: Plastic dynamic viscosity
$$D$$: Diffusion coefficient
$C_{p}$: Specific heat capacity
$$K$$: Thermal conductivity
$$\,\eta$$: Diffusion flux
$B_{0}$: Magnitude of the magnetic field
$$\beta_{\Phi }$$: Concentration coefficient
$\,k$: Debye–Huckel parameter
$k^{\square }$: Relative micro-channel ratio
$${\text{E}}_{{\text{c}}}$$: Eckert number
$Gr$: Thermal grashof number
$$Sc$$: Schmidt number
$${\text{Re}}$$: Reynold number
$$\alpha$$: Fractional order
$$\lambda$$: Time length
$M,\,N$: Number of grid points
$$\overrightarrow {E}$$: Electric field
